# Dependency of Tunneling-Magnetoresistance Ratio on Nanoscale Spacer Thickness and Material for Double MgO Based Perpendicular-Magnetic-Tunneling-Junction

**DOI:** 10.1038/srep38125

**Published:** 2016-12-08

**Authors:** Du-Yeong Lee, Song-Hwa Hong, Seung-Eun Lee, Jea-Gun Park

**Affiliations:** 1 MRAM Center, Department of Electronics and Computer Engineering, Hanyang University, Seoul, 04763, Republic of Korea

## Abstract

It was found that in double MgO based perpendicular magnetic tunneling junction spin-valves *ex-situ* annealed at 400 °C, the tunneling magnetoresistance ratio was extremely sensitive to the material and thickness of the nanoscale spacer: it peaked at a specific thickness (0.40~0.53 nm), and the TMR ratio for W spacers (~134%) was higher than that for Ta spacers (~98%). This dependency on the spacer material and thickness was associated with the (100) body-centered-cubic crystallinity of the MgO layers: the strain enhanced diffusion length in the MgO layers of W atoms (~1.40 nm) was much shorter than that of Ta atoms (~2.85 nm) and the shorter diffusion length led to the MgO layers having better (100) body-centered-cubic crystallinity.

Perpendicular spin-transfer-torque magnetic random access memory (p-STT MRAM) has been intensively researched because of the possibility of it overcoming the scaling limitations of current dynamic random access memory (DRAM) below the 10-nm design rule^1^,^2^. p-STT MRAM cells consist of a selector (i.e., n-MOSFET, mixed-ionic-electronic-conductor, or ovonic-threshold-switch) and a perpendicular-magnetic tunneling junction (p-MTJ) spin-valve^3^,^4^. In general, a p-MTJ spin-valve is fabricated with a vertically stacking structure consisting of a bottom electrode, a seed layer, a synthetic anti-ferromagnetic (SyAF) layer, a magnesium-oxide (MgO) tunneling barrier, a ferromagnetic pinned layer, a ferromagnetic free layer, a capping layer, and a top electrode in which the ferromagnetic free and pinned layer have used CoFeB. A p-MTJ spin-valve has three critical device parameters, i.e., the tunneling magnetoresistance (TMR) ratio, thermal stability factor (Δ), and switching current density (J_C_). The TMR ratio is determined by anti-parallel resistance state (at opposite electron-spin-direction between ferromagnetic free and pinned layer) and parallel resistance state (at same electron-spin-direction between ferromagnetic free and pinned layer), requiring a high TMR ratio of >150% for p-STT MRAM operation. Δ determines the retention time of p-STT MRAM, needing ~74 at 85 °C for 10 years. J_C_ is an extremely critical parameter to achieve low power consumption for p-STT MRAM operation, essentially satisfying <1 MA/cm^2^. In particular, these three critical device parameters should be achieved at the back end of line (BEOL) temperature of 400 °C^5^,^6^,^7^,^8^,^9^, implying that they should not be changed at 400 °C. Note that BEOL means semiconductor processes to integrate p-STT MRAM cells, i.e., wiring, isolation, and passivation etc., requiring the process temperature of ≥400 °C^10^,^11^. Hence, a lot of research has gone into improving these three critical device parameters of p-MTJ spin-valves. In particular, a double MgO based p-MTJ spin-valve has been proposed as a way to enhance thermal stability, although it slightly reduces the TMR ratio^12^,^13^,^14^. In addition, a p-MTJ spin-valve with a top free CoFeB layer rather than a p-MTJ spin-valve with a bottom free CoFeB layer has been studied as a way to achieve a higher TMR ratio at this temperature. Remind that a p-MTJ spin-valve with a top free CoFeB layer has the CoFeB free layer located above the SyAF layer while that with a bottom has the free CoFeB layer located below the SyAF layer. Furthermore, tantalum (Ta) has been used as a spacer material to ferro-couple two ferro-magnetic layers (i.e., the lower CoFeB layer and upper CoFeB layer). However, so far, it has proven extremely difficult achieve a high TMR ratio in a double MgO based p-MTJ spin-valve using a nanoscale thickness Ta spacer at the BEOL temperature of 400 °C^15^,^16^. Moreover, the reason why this is so has not been revealed. Thus, as a solution to this problem, we tried a new spacer material with a body-centered-cubic (bcc) crystal structure (i.e., tungsten). We investigated the dependency of the TMR ratio on the thickness and type of material (tantalum or tungsten) of spacers in double MgO-based p-MTJ spin-valves with a top free Co_2_Fe_6_B_2_ layer. In addition, the mechanism by which thickness and material of the spacer influence the TMR ratio was revealed by examining the static magnetization behavior, (100) bcc crystallinity, and depth profile of the atomic composition of the spin-valves.

## Results

### TMR ratios of p-MTJ spin-valves

**Figure 1:**
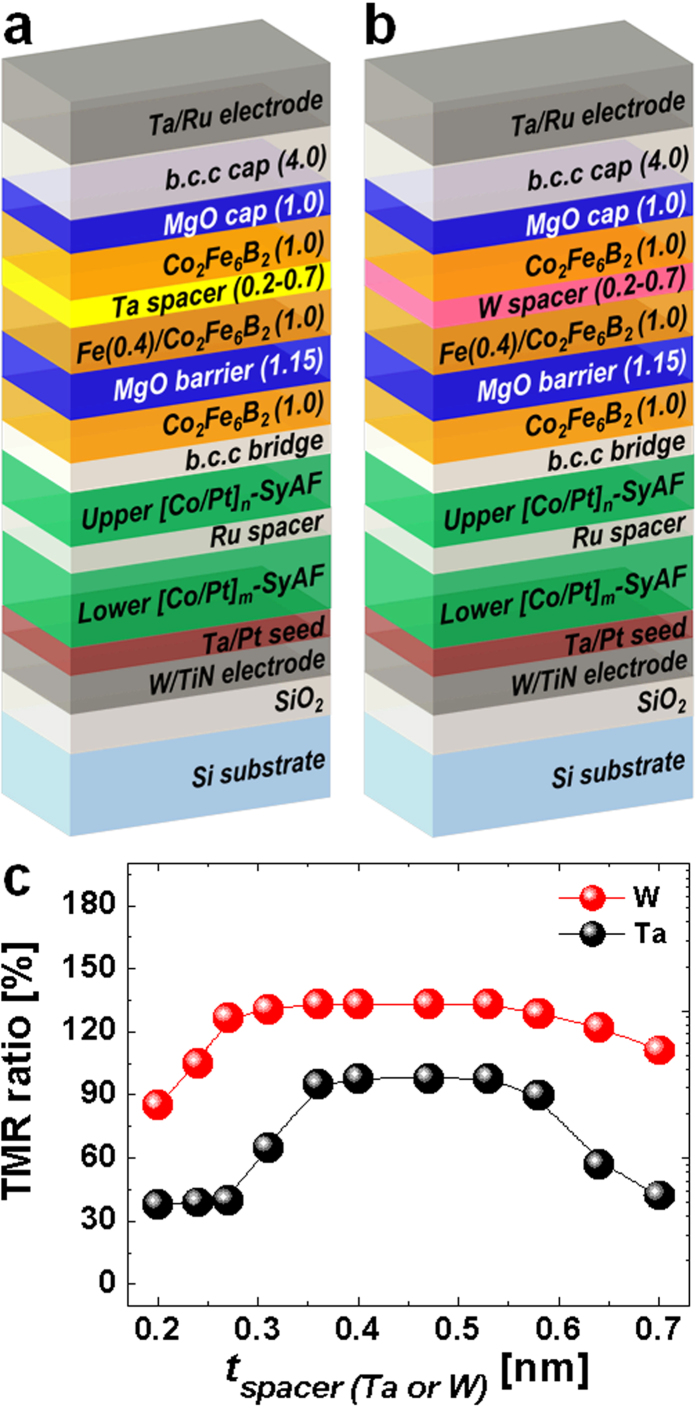
Dependence of TMR ratio on a nanoscale spacer material and thickness for double MgO based p-MTJ spin-valves *ex-situ* annealed at 400 °C for 30 min under perpendicular applied field of 3 Tesla. (**a**) double MgO based on p-MTJ spin-valve using a nanoscale thick Ta spacer, (**b**) double MgO based on p-MTJ spin-valve using a nanoscale thick W spacer, and (**c**) TMR ratio depending on a nanoscale spacer material (Ta or W) and thickness.

The dependence of the TMR ratio on the thickness and type of material of the spacer was estimated by using current-in-plane tunneling (CIPT) technique at room temperature, as shown in Fig. 1c. The TMR ratio can be simply obtained by the CIPT technique without memory-cell patterning requiring a huge process integration; i.e., the four small probes are placed collinearly on a planar tri-layer (top electrode, p-MTJ spin-valve, and bottom electrode), and a current is sent through the planar tri-layer through two probes (I_+_ and I_−_ probes). The resistance of the p-MTJ spin-valve is determined by measuring the induced voltage drop between the other two probes (V_+_ and V_−_) at the 4-probe method^17^. For the Ta spacers, the TMR ratio significantly increased from ~38 to ~95% as the spacer thickness was increased from 0.20 to 0.35 nm. It remained about 98% for spacer thicknesses ranging from 0.40 to 0.53 nm. It decreased from ~90 to ~42% as the spacer thickness was further increased from 0.58 to 0.70 nm. Thus, the TMR ratio (~98%) peaked in a certain nanoscale spacer thickness region (0.40~0.53 nm) for Ta. Next, regarding the samples made with W spacers, the TMR ratio significantly increased from ~85 to ~131% as the spacer thickness was increased from 0.20 to 0.30 nm. It remained at ~134% as the spacer thickness was further increased from 0.40 to 0.53 nm. The ratio slightly decreased from ~134 to ~111% for larger thicknesses ranging from 0.58 to 0.70 nm. Thus, the TMR ratio (~134.0%) also peaked in a specific nanoscale spacer thickness region (0.40~0.53 nm) for W. Comparing the TMR ratios in the cases of the Ta spacer and the W spacer obviously indicates that the use of 0.20 and 0.70 nm-thick W spacers resulted in least 35% higher TMR ratios in the comparison with the use of Ta spacers. In particular, both the thinnest (0.20 nm) and thickest (0.70 nm) W spacers yielded much higher TMR ratios (~85 and ~111%) in comparison with the corresponding Ta spacers (~38 and ~42%).

### Magnetic properties of p-MTJ spin-valves

**Figure 2:**
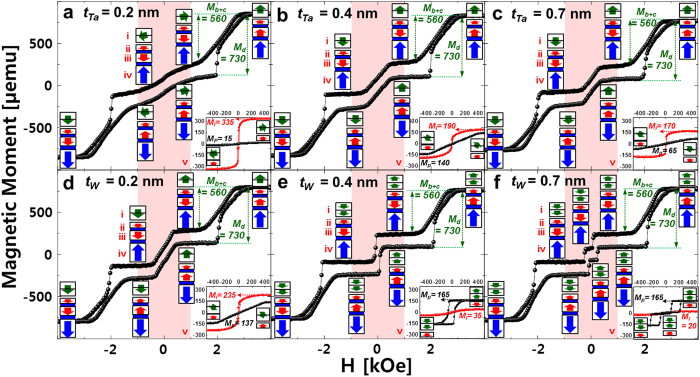
Dependence of M-H curves on a nanoscale spacer material and thickness for double MgO based p-MTJ spin-valves *ex-situ* annealed at 400 °C for 30 min under perpendicular applied field of 3 Tesla. M-H curve of (**a**) t_Ta_ = 0.2 nm, (**b**) t_Ta_ = 0.4 nm, (**c**) t_Ta_ = 0.7 nm, (**d**) t_W_ = 0.2 nm, (**e**) t_W_ = 0.4 nm, and (**f**) t_W_ = 0.7 nm. The red shadow regions in Fig. (**a–f**) point out the static magnetization behavior of the Co_2_Fe_6_B_2_ free layer ferro-coupled with the upper Co_2_Fe_6_B_2_ free layer. The insets of Fig. (**a–f**) are M-H curves of the Co_2_Fe_6_B_2_ free layer ferro-coupled with the upper Co_2_Fe_6_B_2_ free layer.

To reveal the reason why the TMR ratio peaked at a specific range of nanoscale spacer thicknesses, the dependency of the static magnetization behavior of double MgO based p-MTJ spin-valves on the spacer thickness and spacer material was investigated by measuring the magnetic moment vs. magnetic field (M-H) curves using a vibrating sample magnetometer (VSM) at room temperature (Fig. 2). VSM is a tool to measure M-H curves under perpendicular or in-plane magnetic field through probe vibration. The static magnetization behavior for the cut samples fabricated with the p-MTJ spin-valve structure was analyzed with a VSM at room temperature. The two spin-valves consisted of four magnetic layers, as shown in Fig. 2a–f: a Co_2_Fe_6_B_2_ free layer ferro-coupled with the upper Co_2_Fe_6_B_2_ free layer via the Ta or W spacer (**i**), a Co_2_Fe_6_B_2_ pinned layer (**ii**), an upper SyAF layer (**iii**), and a lower SyAF layer (**iv**); the lengths of boxes and vectors in the figure correspond to the magnitude of the magnetic moment and spin-electron direction for a magnetic layer. Initially, the Co_2_Fe_6_B_2_ pinned layer (**ii**) was ferro-coupled with the upper SyAF layer (**iii**), while the upper SyAF layer (**iii**) was anti-ferro-coupled with the lower SyAF layer (**iv**)^15^,^18^,^19^. The SyAF layer in a p-MTJ spin-valve is essentially necessary to fixing the electron-spin-direction of the Co_2_Fe_6_B_2_ pinned layer by ferro-coupling with the lower SyAF layer. Thus, the M-H curves in Fig. 2a–f correspond to the static magnetization behavior of the double MgO based p-MTJ spin-valves when the applied magnetic field was scanned from 0, 4, 0, and −4 to 0 kOe, while the M-H curve in insets of Fig. 2a–f correspond to the static magnetization behavior of only the Co_2_Fe_6_B_2_ free layer ferro-coupled with the upper Co_2_Fe_6_B_2_ free layer via the Ta or W spacer when the applied magnetic field was scanned through 0, 500, 0, and −500 to 0 Oe.

First, regarding the spin-valve with the Ta spacer, the perpendicular magnetic anisotropy (PMA) characteristics of the (Co/Pt)_n_-SyAF layer and interfacial-perpendicular magnetic anisotropy (i-PMA)^20^,^21^ characteristics of the Co_2_Fe_6_B_2_ pinned layer did not vary when the spacer thickness was varied from 0.2 to 0.7 nm; i.e., M_d_ (730 μemu) and M_b+c_ (560 μemu) were almost constant despite the change in Ta spacer thickness, as shown in Fig. 2a–c. In contrast, the i-PMA characteristics of the Co_2_Fe_6_B_2_ free layer ferro-coupled with the upper Co_2_Fe_6_B_2_ free layer depended considerably on the spacer thickness (t_Ta_). At t_Ta_ = 0.20 nm, the Co_2_Fe_6_B_2_ free layer ferro-coupled with the upper Co_2_Fe_6_B_2_ free layer via the Ta spacer had almost in-plane magnetic anisotropy (IMA); the IMA magnetic moment (M_I_) and i-PMA magnetic moment (M_p_) of the Co_2_Fe_6_B_2_ free layer ferro-coupled with the upper Co_2_Fe_6_B_2_ free layer via the Ta spacer were 335 and 15 μemu, respectively, as shown in **v** in Fig. 2a and the inset. Note that PMA comes from bulk perpendicular ferromagnetic layer [i.e, (Co/Pt)_n_-SyAF layer] while i-PMA originates form the interface between the MgO tunneling barrier and the Co_2_Fe_6_B_2_ ferromagnetic layer. The p-MTJ spin-valve had an extremely low TMR ratio (~38%) because the Co_2_Fe_6_B_2_ free layer would be bridged with the upper Co_2_Fe_6_B_2_ free layer due to Ta diffusion during *ex-situ* annealing at 400 °C; the magnetic properties of the Co_2_Fe_6_B_2_ free layer transformed from i-PMA to IMA. Note that the TMR ratio of double MgO based p-MTJ spin-valves is determined mostly by M_p_; i.e., a higher M_p_ leads to a higher TMR ratio. By comparison, at t_Ta_ = 0.40 nm, the Co_2_Fe_6_B_2_ free layer ferro-coupled with the upper Co_2_Fe_6_B_2_ free layer via the Ta spacer exhibited a mixture of IMA and i-PMA characteristics because the 0.4-nm-thick Ta spacer would sufficiently separate and ferro-couple the Co_2_Fe_6_B_2_ free layer with the upper Co_2_Fe_6_B_2_ free layer, where M_I_ and M_p_ were 190 and 140 μemu, respectively, as shown in **v** in Fig. 2b and the inset. When t_Ta_ increased from 0.20 nm to 0.40 nm, M_I_ considerably decreased from 335 to 190, while M_p_ remarkably increased from 15 to 140 μemu. However, at t_Ta_ = 0.70 nm, the Co_2_Fe_6_B_2_ free layer ferro-coupled with the upper Co_2_Fe_6_B_2_ free layer via the Ta spacer also showed a mixture of IMA and i-PMA characteristics, where M_I_ and M_p_ were 170 and 65 μemu, respectively, as shown in **v** in Fig. 2c and the inset. As t_Ta_ increased from 0.40 nm to 0.70 nm, M_I_ slightly decreased from 190 to 170 μemu, while M_p_ greatly decreased from 140 to 65 μemu. Therefore, for the double MgO based p-MTJ spin-valve with the Ta spacer, the dependency of M_p_ on t_Ta_ was correlated with that of the TMR ratio on t_Ta_; i.e., both dependencies peak a specific nanoscale t_Ta_.

Second, for the spin-valve with the W spacer, the PMA characteristics of the (Co/Pt)_n_-SyAF layer and i-PMA characteristics of the Co_2_Fe_6_B_2_ pinned layer did not change when the spacer thickness was changed from 0.2 to 0.7 nm; i.e., M_d_ (730 μemu) and M_b+c_ (560 μemu) were almost constant despite the change in spacer thickness, as was the case with the spin-valve with the Ta spacer, as shown in Fig. 2d–f. However, the i-PMA characteristics of the Co_2_Fe_6_B_2_ free layer ferro-coupled with the upper Co_2_Fe_6_B_2_ free layer via the W spacer remarkably depended on t_W_. At t_W_ = 0.20 nm, the Co_2_Fe_6_B_2_ free layer showed a mixture of IMA and i-PMA characteristics, where M_I_ and M_p_ were 235 and 137 μemu, respectively, resulting in a low TMR ratio, as shown in **v** in Fig. 2d and the inset. A comparison of Fig. 2a with Fig. 2d obviously implies that M_p_ in the case of the W spacer at t_W_ = 0.20 nm (137 μemu) was higher than that at t_Ta_ = 0.20 nm (15 μemu), suggesting that the TMR ratio at t_W_ = 0.20 nm is higher than at t_Ta_ = 0.20 nm. In addition, as t_W_ increased from 0.20 to 0.40 nm, M_p_ considerably increased from 137 to 165 μemu, while M_I_ greatly decreased from 235 to 35 μemu, suggesting a TMR ratio increase (see the inset of Fig. 2e). Note that the M_p_ of 165 μemu is an ideal value for the Co_2_Fe_6_B_2_ free layer ferro-coupled with the upper Co_2_Fe_6_B_2_ free layer via the W spacer since the M_p_ of only the Co_2_Fe_6_B_2_ free layer without a ferro-coupling with the upper Co_2_Fe_6_B_2_ free layer is ~83 μemu. A comparison of Fig. 2b with Fig. 2e clearly indicates that the M_p_ of the Co_2_Fe_6_B_2_ free layer ferro-coupled with the upper Co_2_Fe_6_B_2_ free layer via the W spacer at t_W_ = 0.40 nm (165 μemu) was higher than that at t_Ta_ = 0.40 nm (140 μemu), suggesting that the TMR ratio at t_W_ = 0.40 nm is higher than that at t_Ta_ = 0.40 nm. However, when t_W_ increased from 0.40 to 0.70 nm, M_p_ remained at ~165 μemu, while M_I_ slightly decreased from 35 to 20 μemu, suggesting almost no change in the TMR ratio (see the inset of Fig. 2f). However, the TMR ratio decreased from ~134% to ~111% when t_W_ increased from 0.40 to 0.70 nm, which is probably associated with the (100) bcc crystallinity degradation of the MgO tunneling barrier when t_W_ increased from 0.40 to 0.70 nm. In particular, the M-H curve in the inset of Fig. 2f evidently shows that the Co_2_Fe_6_B_2_ free layer was not completely ferro-coupled with the upper Co_2_Fe_6_B_2_ free layer via the W spacer. A comparison of Fig. 2c with Fig. 2f obviously implies that M_p_ at t_W_ = 0.70 nm (165 μemu) was higher than that at t_Ta_ = 0.70 nm (65 μemu) and that the TMR ratio at t_W_ = 0.70 nm is higher than that at t_Ta_ = 0.70 nm. Therefore, for the spin-valve with the W spacer, the dependency of M_p_ on t_W_ is not correlated with that of the TMR ratio on t_W_, which would be related to the dependency of the crystallinity of the MgO tunneling-barrier on t_W_.

### Crystallinity of the MgO tunnel barrier

**Figure 3:**
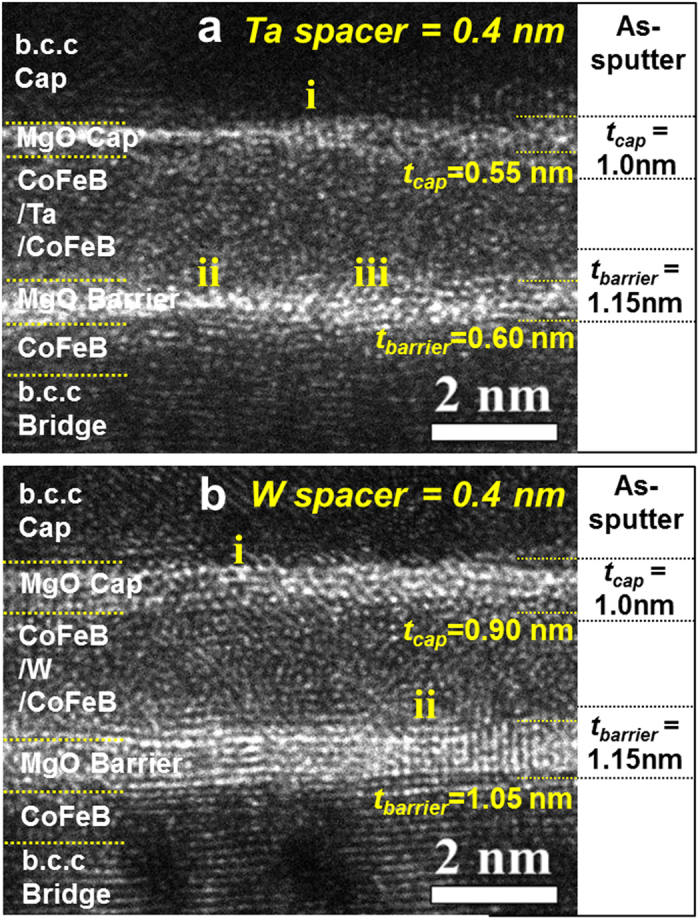
Dependence of the (100) b.c.c crystallinity of the capping MgO layer and the MgO tunneling barrier on a nanoscale spacer material for double MgO based p-MTJ spin-valves *ex-situ* annealed at 400 °C for 30 min under perpendicular applied field of 3 Tesla. x-HR-TEM images of double MgO based p-MTJs for (**a**) Ta spacer of 0.4 nm and (**b**) W spacer of 0.4 nm.

The TMR ratio for the p-MTJ spin-valves is mainly determined by the i-PMA characteristics (i.e., M_p_) of the Co_2_Fe_6_B_2_ free layer ferro-coupled with the upper Co_2_Fe_6_B_2_ free layer via the spacer as well as on the bcc crystallinity of the MgO tunneling barrier^22^. In order to reveal reason the M_p_ and TMR ratio were higher for the W spacer at an *ex-situ* annealing temperature of 400 °C, the dependency of the bcc crystallinity of the MgO-tunneling-barrier on the thickness of the spacer material (Ta or W) was investigated via a cross-sectional high-resolution transmission-electron-microscopy (x-HR-TEM) at 200 keV, as shown Fig. 3. Note that the p-MTJ consisted of a vertically stacked structure: MgO tunneling barrier (1.15 nm)/Fe (0.40 nm)/Co_2_Fe_6_B_2_ free layer (1.0 nm)/spacer (Ta or W: 0.2~0.7 nm)/upper Co_2_Fe_6_B_2_ free layer (1.0 nm)/ MgO capping layer (1.0 nm). The MgO capping layer in the spin-valve with the Ta spacer was revealed to be amorphous (**i** in Fig. 3a) and its thickness was ~0.55 nm; ~0.45 nm had been consumed due to Ta atom diffusion during the *ex-situ* annealing. In addition, the MgO tunneling barrier was revealed to be a mixture of an amorphous layer (**ii** in Fig. 3a) and a (100) body-centered-cubic (b.c.c) crystallized layer (**iii** in Fig. 3a)^23^,^24^,^25^, and its thickness was ~0.60 nm; ~0.55 nm was consumed by diffusion during the *ex-situ* annealing. The thickness of consumed part of the MgO capping layer was similar to that of the MgO tunneling barrier since the Ta atoms from the Ta spacer diffused in both directions toward the capping layer and tunneling barrier during the annealing. By comparison, the spin-valve with the W spacer had a slightly (100) crystallized MgO capping layer (**i** in Fig. 3b) and its thickness was ~0.90 nm; ~0.10 nm was consumed through W diffusion during annealing. In addition, the MgO tunneling barrier had a well (100) crystallized layer (**ii** in Fig. 3b), and its thickness was ~1.05 nm (~0.10 nm was consumed through W atom diffusion during annealing). Also, the thickness of the consumed part of the MgO capping layer was similar to that of the MgO tunneling barrier. A comparison of Fig. 3a with Fig. 3b evidently indicates that the (100) bcc crystallinities of the MgO capping layer and tunneling barrier with the W spacer were superior to those of the spin-valve with the Ta spacer. In particular, the thickness of the consumed parts of the MgO capping layer and the MgO tunneling barrier was much less in the case of the W spacer (~0.10 nm) than in the case of the Ta spacer (~0.55 nm). Since the (100) bcc crystallinity of the MgO tunneling barrier greatly influences the TMR ratio, the HR-TEM results in Fig. 3 obviously show why the TMR ratio was much higher in the case of the W spacer (~134%), i.e., from the difference between the (100) bcc crystallinities of the MgO tunneling barriers in the cases of 0.4 nm Ta and W spacers. In addition, since the i-PMA magnetic moment of the Co_2_Fe_6_B_2_ free layer ferro-coupled with the upper Co_2_Fe_6_B_2_ free layer via the W spacer (M_p_) was also greatly influenced by the (100) bcc crystallinities of both the capping layer and tunneling barrier, the HR-TEM results in Fig. 3 evidently explain why the M_p_ of the spin-valve was higher with the W spacer (~165 μemu) than with the Ta spacer (~140 μemu) at the spacer thickness of 0.4 nm. Furthermore, the correlation of the results in Fig. 2b and e with those of Fig. 3a and b clearly implies that the W spacer resulted in much better (100) bcc crystallinities of both the capping layer and the tunneling barrier at the *ex-situ* annealing temperature of 400 °C, and thus, the M_p_ in the case of the W spacer was higher than in the case with the Ta spacer and caused the TMR ratio for the W spacer to be higher than that of the Ta spacer^26^,^27^.

### Atomic compositional depth profile

**Figure 4:**
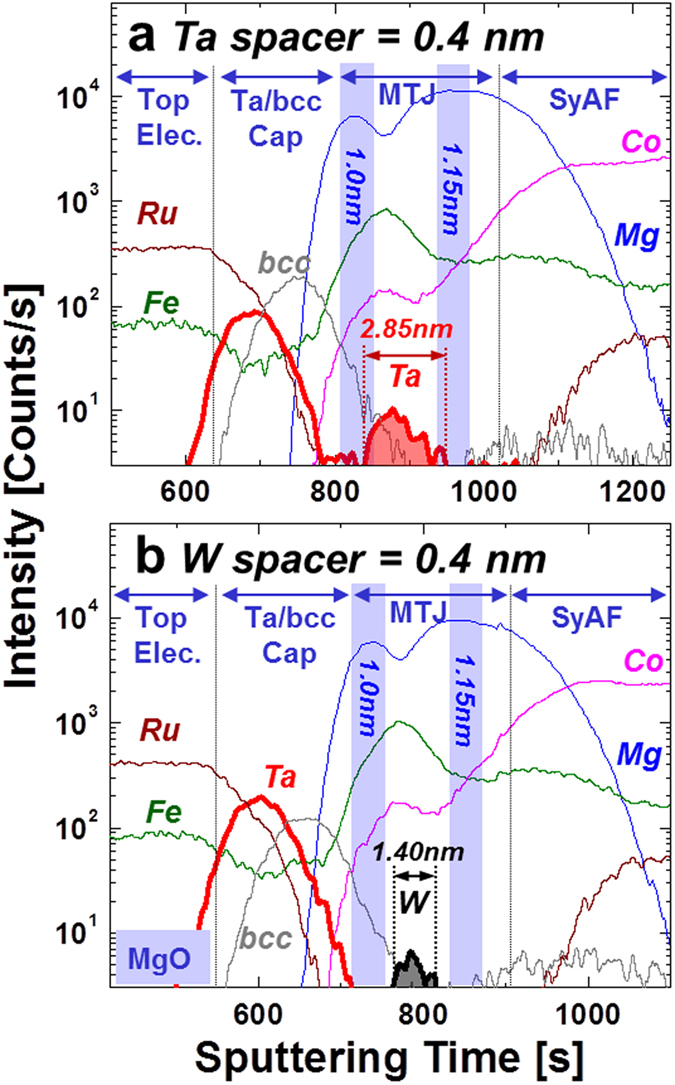
Dependence of the atomic compositional depth profiles on a nanoscale spacer material for double MgO based p-MTJ spin-valves *ex-situ* annealed at 400 °C for 30 min under perpendicular applied field of 3 Tesla. (**a**) Ta spacer of 0.4 nm and (**b**) W spacer of 0.4 nm.

To determine why a W spacer results in capping layers and tunneling barriers with much better (100) bcc crystallinity, the dependence of the atomic compositional depth profile of the spin-valves on the spacer material (W or Ta) was investigated by using high-resolution secondary-ion-mass-spectroscopy (HR-SIMS: sputtering rate of 0.5 Å/sec). As shown in Fig. 4a and b, the atomic compositional depth profiles except for the spacer (Ta or W) were the same. For the Ta spacer, the diffusion length of Ta atoms during an *ex-situ* annealing at 400 °C was ~2.85 nm and the relative Ta concentration was 10 counts, indicating that Ta atoms diffused completely toward both the upper Co_2_Fe_6_B_2_ free layer/the MgO capping layer and the Co_2_Fe_6_B_2_ free layer/the MgO tunneling layer. Thus, both the capping layer and tunneling barrier were consumed by diffusion of Ta atoms such that they had worse (100) bcc crystallinity (they had an amorphous layer or a mixture of amorphous and a bcc crystallized layer; compare Figs 3a and 4a). On the other hand, for the W spacer, the diffusion length was ~1.40 nm, and the relative concentration was 6 counts, indicating that W atoms diffused only to edges of the upper and lower free layers since the sum thickness of the upper Co_2_Fe_6_B_2_ layer (~1.0 nm), W spacer (~0.4 nm), and lower Co_2_Fe_6_B_2_ free layer (~1.0 nm) prior to the *ex-situ* annealing was ~2.4 nm. Thus, the capping layer and tunneling barrier were not consumed by the diffusion of W atoms and showed good (100) bcc crystallinity (compare Figs 3b and 4b). In summary, the reason why the W spacer yielded much better (100) bcc crystallinity for both the capping layer and tunneling barrier is that the diffusion length (~1.40 nm) was much shorter than that for the Ta spacer (~2.85 nm).

### Strain at the interface between free layer and spacer

**Figure 5:**
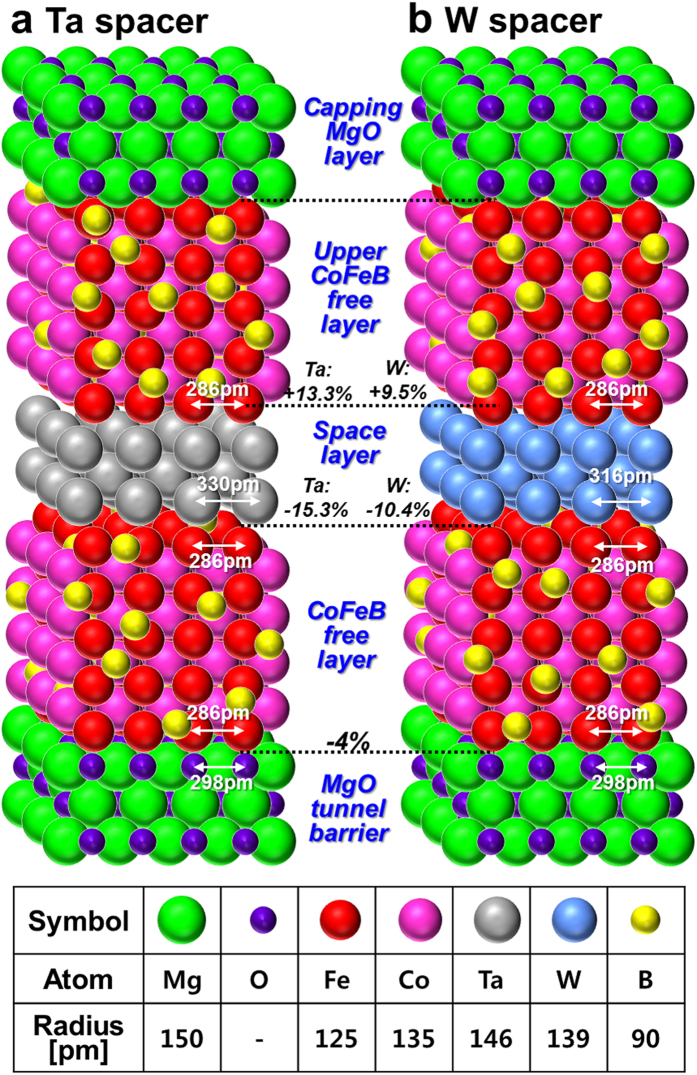
Atomic structure of double MgO based p-MTJs. Nanoscale thickness (**a**) Ta and (**b**) W spacers for double MgO based p-MTJs. The table lists the atomic sizes.

The reason the diffusion length of W atoms (~1.40 nm) in the p-MTJ was much shorter than that of Ta atoms (~2.85 nm) would be related to strained enhanced atomic diffusion at the interface with the Co_2_Fe_6_B_2_ layer and at the interface with the upper Co_2_Fe_6_B_2_ free layer. The strains at these interfaces can be calculated as follows: [(a_CFB _− a_Ta or W_)/a_CFB_] × 100% or [(a_Ta or W_ − a_CFB_)/a_Ta or W_] × 100%, where a_CFB_ and a_Ta or W_ are the lattice constants of the Co_2_Fe_6_B_2_ layer and the Ta or W spacer, respectively. Note that the i-PMA characteristics originate from hybridization of Fe_3d_-O_2p_ and Co_3d_-O_2p_ at the interface, where an amorphous Co_2_Fe_6_B_2_ layer is textured with (100) bcc MgO layer during *ex-situ* annealing at 400 °C^28^,^29^,^30^,^31^. In addition, the lattice constants of Ta, W, and the Co_2_Fe_6_B_2_ layer are 330, 316, and 286 pm, respectively^32^,^33^. Thus, the strain at the interface between the free layer and Ta spacer was −15.3%, and the strain at the interface between the Ta spacer and the upper layer was +13.3%, where “−” means compressive strain and “+” means tensile strain, as shown in Fig. 5a. By comparison, the corresponding strains at these interfaces with the W spacer were −10.4% and +9.5%, as shown in Fig. 5b. These results obviously indicate that the strains at the interfaces of the Co_2_Fe_6_B_2_ free layer and the spacer with the Ta spacer were much larger than those with the W spacer. During *ex-situ* annealing at 400 °C for 30 min under an applied vertical magnetic field of 3 Tesla, the diffusivity of Ta or W atoms into the Co_2_Fe_6_B_2_ free layer/MgO layer would be enhanced by the strains at the interfaces between the spacers and the Co_2_Fe_6_B_2_ layer. The strain enhanced diffusion is defined as D(σ) = D_0_exp[(−E_a_ + Ωσ)/kT], where D_0_, E_a_, Ω, σ are the diffusivity without considering the strain effect, diffusion activation energy, atomic volume, and scalar hydrostatic stress, respectively^34^,^35^. In addition, the atomic radius of Ta and W atoms are 146 and 139 pm, respectively^36^. The diffusivity of Ta atoms into the Co_2_Fe_6_B_2_ free layer/MgO layer would be much larger than that of W atoms since the Ta atomic volume and the strains at the interface between the Ta spacer and the Co_2_Fe_6_B_2_ free layer are much larger than the W atomic volume and the corresponding strain at the interface. Thus, the diffusion length of Ta atoms into the Co_2_Fe_6_B_2_ free layer/MgO layer would be much longer than that of W atoms, as shown in Fig. 4. Note that the diffusion length is proportional to the square root of diffusivity. In summary, the reason why the diffusion length of W atoms (~1.40 nm) in the p-MTJ was much shorter than that of Ta atoms in the p-MTJ (~2.85 nm) is that the strain enhanced diffusivity of Ta atoms into the Co_2_Fe_6_B_2_ and MgO layers was much larger than that of W atoms.

## Discussion

In summary, for double MgO based p-MTJ spin-valves, the material and thickness of the nanoscale spacer for ferro-coupling the Co_2_Fe_6_B_2_ free layer with the upper Co_2_Fe_6_B_2_ layer play a critical role in achieving a higher TMR ratio at a higher BEOL temperature in p-STT MRAM integration (i.e., 400 °C). The maximum TMR ratio occurs in a specific range of spacer thicknesses (i.e., 0.40~0.53 nm) and is much higher for a W spacer (~134%) than for a Ta spacer (~98%). These properties are related to the (100) bcc crystallinity of the MgO tunneling barrier and capping layer: the crystallinities are much better when a W spacer is used instead of a Ta spacer. Consequently, the PMA magnetic moment of the Co_2_Fe_6_B_2_ free layer ferro-coupled with the upper Co_2_Fe_6_B_2_ free layer via the W spacer was higher than that with a Ta spacer. This difference in crystallinity between using W and Ta spacer for the capping layer and tunneling barrier at an *ex-situ* annealing temperature at 400 °C is related to the diffusion length of the spacer atoms into the Co_2_Fe_6_B_2_ layer/MgO layer: the diffusion length is much shorter for W atoms (~1.40 nm) than for Ta (~2.85 nm) and the short W diffusion length leads to the MgO capping layer and tunneling barrier having better (100) bcc crystallinities. This diffusion length difference is associated with a difference in strain enhanced diffusivity into the Co_2_Fe_6_B_2_ layer/MgO layer: the strain at the interfaces between the Co_2_Fe_6_B_2_ layer and the spacer with a W spacer is much less than that for a Ta spacer and hence the strain enhanced diffusivity of Ta atoms into the Co_2_Fe_6_B_2_ layer/MgO layer is much larger than that of W atoms. Therefore, for double MgO based p-MTJ spin-valves, the material and thickness of the spacer are important design considerations to minimize the interface strain between the nanoscale thick spacer and the Co_2_Fe_6_B_2_ layers and achieve the maximum TMR ratio and thermal stability at the BEOL of 400 °C in p-STT MRAM integration.

## Methods

Two types of double MgO based p-MTJ spin-valves with a [Co/Pt]_n_-based SyAF layer were fabricated on a 12-inch-diameter wafer deposited with SiO_2_/W (160 nm)/TiN (40 nm) films in a 12-inch multi-chamber sputtering system under a high vacuum of less than 1 × 10^−8^ torr without breaking the vacuum. As shown in Fig. 1a and b, p-MTJ spin-valves with a nanoscale thick Ta or W spacer were fabricated with vertically stacked layers of TiN (40 nm) bottom electrode/Ta buffer layer (5.0 nm)/Pt (3.0 nm) seed layer/lower [Co (0.4 nm)/Pt (0.3 nm)]_6_-SyAF layer/Co (0.4 nm)/Ru spacer (0.85 nm)/upper [Co (0.4 nm)/Pt (0.3 nm)]_5_-SyAF layer/Co (0.4 nm)/b.c.c (0.2 nm) bridge layer/Co_2_Fe_6_B_2_ (1.0 nm) pinned-layer/MgO (1.15 nm) tunnel barrier/[Fe (0.4 nm)/Co_2_Fe_6_B_2_ (1.0 nm)/spacer (Ta or W: 0.2~0.7 nm)/Co_2_Fe_6_B_2_ (1.0 nm)] free-layer/[MgO (1.0 nm)/bcc (4.0 nm)] capping layer/Ta/Ru top electrode. Note that the 12-inch wafers were subject to chemical-mechanical-planarization process just after TiN electrode deposition to achieve the surface roughness (rms) of ~2 Å. The bcc bridging and capping layer was made by W. All samples were subject to *ex-situ* annealing at 400 °C for 30 min under a perpendicular magnetic field of 3 Tesla (i.e., 30000 Gauss). In addition, after CIPT measurement, 12-inch wafers fabricated with the p-MTJ spin-valve structure were cut into 1 × 1 cm^2^ pieces. The static magnetization behavior for the cut samples fabricated with the p-MTJ spin-valve structure was analyzed with a vibrating sampling magnetometer (VSM) at room temperature. The crystallinity of the MgO tunneling barrier was investigated by cross-sectional high-resolution transmission-electron-microscopy (x-HR-TEM) observations at 200 keV in acceleration voltage, the atomic compositional depth profile by using high-resolution secondary-ion-mass-spectroscopy (HR-SIMS: sputtering rate of 0.5 Å/sec), and the strain at the interface between the free layer and spacer by theoretical calculation.

## Additional Information

**How to cite this article**: Lee, D.-Y. *et al*. Dependency of Tunneling-Magnetoresistance Ratio on Nanoscale Spacer Thickness and Material for Double MgO Based Perpendicular-Magnetic-Tunneling-Junction. *Sci. Rep.* **6**, 38125; doi: 10.1038/srep38125 (2016).

**Publisher's note:** Springer Nature remains neutral with regard to jurisdictional claims in published maps and institutional affiliations.
